# Contribution of the one health approach to strengthening health security in Uganda: a case study

**DOI:** 10.1186/s12889-023-15670-3

**Published:** 2023-08-07

**Authors:** Herbert Bakiika, Ekwaro A. Obuku, Justine Bukirwa, Lydia Nakiire, Aruho Robert, Maureen Nabatanzi, Mwebe Robert, Mwanja Moses, Martha Isabella Achan, John Baptist Kibanga, Aisha Nakanwagi, Issa Makumbi, Immaculate Nabukenya, Mohammed Lamorde

**Affiliations:** 1 Infectious Diseases Institute, Makerere University, P.O. Box 22418, Kampala, Uganda; 2 Public Health Emergency Operation Centre, Ministry of Health, P.O. Box 7272, Kampala, Uganda; 3 Ministry of Agriculture and Animal Industry and Fisheries, P.O Box 102, Entebbe, Uganda; 4 Uganda Wildlife Authority, P.O Box 3530, Kampala, Uganda

**Keywords:** One health, International Health Regulations, Global Health Security, National Action Plan for Health Security

## Abstract

**Background:**

The One Health approach is key in implementing International Health Regulations (IHR, 2005) and the Global Health Security Agenda (GHSA). Uganda is signatory to the IHR 2005 and in 2017, the country conducted a Joint External Evaluation (JEE) that guided development of the National Action Plan for Health Security (NAPHS) 2019–2023.

**Aim:**

This study assessed the contribution of the One Health approach to strengthening health security in Uganda.

**Methods:**

A process evaluation between 25th September and 5th October 2020, using a mixed–methods case study. Participants were Subject Matter Experts (SMEs) from government ministries, departments, agencies and implementing partners. Focus group discussions were conducted for five technical areas (workforce development, real-time surveillance, zoonotic diseases, national laboratory systems and emergency response operations), spanning 18 indicators and 96 activities. Funding and implementation status from the NAPHS launch in August 2019 to October 2020 was assessed with a One Health lens.

**Results:**

Full funding was available for 36.5% of activities while 40.6% were partially funded and 22.9% were not funded at all. Majority (65%) of the activities were still in progress, whereas 8.6% were fully implemented and14.2% were not yet done. In workforce development, several multisectoral trainings were conducted including the frontline public health fellowship program, the One Health fellowship and residency program, advanced field epidemiology training program, in-service veterinary trainings and 21 district One Health teams’ trainings. Real Time Surveillance was achieved through incorporating animal health events reporting in the electronic integrated disease surveillance and response platform. The national and ten regional veterinary laboratories were assessed for capacity to conduct zoonotic disease diagnostics, two of which were integrated into the national specimen referral and transportation network. Multisectoral planning for emergency response and the actual response to prioritized zoonotic disease outbreaks was done jointly.

**Conclusions:**

This study demonstrates the contribution of ‘One Health’ implementation in strengthening Uganda’s health security. Investment in the funding gaps will reinforce Uganda’s health security to achieve the IHR 2005. Future studies could examine the impacts and cost-effectiveness of One Health in curbing prioritized zoonotic disease outbreaks.

## Introduction

Globally, over 75% of emerging and re-emerging infections in humans originate from animals [1]. In fact, HIV/AIDS discovered around 1980 and COVID-19 about four decades later, are two global pandemics that are thought to have origins in animals [2]. Together, these two have caused 40 million and 6 million total deaths respectively [3, 4]. In addition, the FAO predicted that increasing population pressure in Africa results into closer interaction between humans, animals and environment thus higher risk of zoonoses [5].

One Health is a worldwide paradigm shift for expanding interdisciplinary, multisectoral collaborations and communications in all aspects of health care for animals, the environment and humans interface at subnational, national, global, and regional levels [1, 6]. Zoonotic diseases, food borne diseases, chemical events, radiological events, and antimicrobial resistance are complex, and could not be managed by the human health sector single-handedly [1]. The World Health Organization (WHO), Food and Agricultural Organization of the United Nations (FAO),World Organization for Animal Health (OIE) and now United Nations Environment Program recognize the One Health approach in addressing health threats at the interface of human, animal and environment [7]. In 2010, FAO, OIE and WHO signed a tripartite agreement [1] to strengthen multisectoral collaboration to achieve seventeen sustainable development goals to transform our world. Some include; goal one on ending poverty, goal two on ending hunger, goal three on good health and well-being, goal 6 on access to clean water and sanitation, goal 12 on responsible consumption and production, goal 13 on climate action and goal 14 on life below water [8]. In 2022, this formally became the Quadripartite when the United Nations Environment Programme (UNEP) joined and a new Memorandum of Understanding was signed by all four parties [7].

Following the quadripartite, there was development of the one health joint plan of action (OH JPA) which outlines the commitment of the four organizations to collectively advocate and support the implementation of One Health. It builds on, complements, and adds value to existing global and regional One Health and coordination initiatives aiming at strengthening capacity to address complex multidimensional health risks with more resilient health systems at global, regional and national levels [9].

Uganda piloted the Global Health Security Agenda (GHSA) in 2014 and conducted the Joint External Evaluation (JEE) in 2017, both aimed at assessing the country’s progress in strengthening the IHR 2005 core capacities. This resulted into development of the National Plan for Health Security (NAPHS) 2019–2023 under the guidance of the Office of the Prime Minister [10]. NAPHS was launched to strengthen Uganda’s health security capacity and community resilience against public health threats in compliance with IHR 2005. The guiding principles of the NAPHS 2019–2023 are four, namely: One Health approach, multi-sectoral approach, collective responsibility, and collaboration and partnerships [10]. However, to date, there is scarcity of information on the contribution of the ‘One Health’ approach to the progress of implementation of health security. Accordingly, this paper describes the contribution of ‘One Health’ to health security in Uganda. This evidence is imperative in improving health security in Uganda and similar settings globally [10].

## Methods and materials

### Study design

This was a process evaluation using a mixed methods case study. This design was appropriate because it enabled description, exploration and explanation of the phenomenon [11]. Focused Group discussions involving subject matter experts per technical area were conducted and triangulated findings with information from the desk reviews. Commonalities were established on the progress of the NAPHS implementation in Uganda from these two information sources. Five technical areas were assessed for funding status and progress of implementation. Preliminary results were validated by 43 resource persons from various ministries, departments and agencies (MDAs), and implementing partners.

### Study setting and program description

The study period was from September 25th to October 06th 2020 following annual review of the NAPHS implementation. This study was conducted in Uganda which is located in East Africa, West of Kenya, South of South Sudan, East of Democratic Republic of Congo, and North of Rwanda and Tanzania. The country lies between latitude 1^o^ 22’ 14.63’ N and longitude 32^o^ 18’ 11.67’ E, in the Congo basin, making it a hotspot for emerging and re-emerging outbreaks of zoonotic and vector-borne diseases like Ebola, Anthrax, Marburg, Yellow fever [10]. Uganda’s vision 2040 aspires for healthy, wealthy and resilient communities and therefore seeks to combat biological threats and health emergencies [12]. As such, Uganda is signatory to the International Health Regulations (IHR) 2005, which mandates member states to strengthen capacities for health security [13]. Achievement of health security is important in strengthening country’s capacity while aligning to Global Health Security Agenda (GHSA) action packages of prevention, detection and responding to public health emergencies [14].

### Study population

The study population consisted of technical leads from different MDAs (Additional File.1) as key informants in zoonotic diseases, national laboratory systems, real–time surveillance, workforce development and emergency response. These are five of the nineteen technical areas in the NAPHS. Additional participants in this study were secretariat members of the National One Health Platform (NOHP) with a responsibility of oversight and coordination of the One Health approach in Uganda.

### Variables and measurements

The outcome variable in this study is health security. In this study, health security was defined as the activities required, both proactive and reactive, to minimize the danger and impact of acute public health events that endanger people’s health across geographical regions and international boundaries and in this study, we considered health security as the ability of the country to prevent, detect and respond to public health emergencies. Health security was measured using five technical areas with respective indicators namely zoonotic diseases, national laboratory systems, surveillance, workforce development and emergency response as indicated in the IHR 2005 (Table 1) [15]. In addition, governance structures, multisectoral communication and capacity building for the One Health approach were also assessed.

The definition of ‘One Health’ was operationalized as strengthened multisectoral communication, coordination and collaboration across four-line ministries of Ministry of Agriculture, Animal Industries and Fisheries, Uganda Wildlife Authority, Ministry of water and Environment and Ministry of Health.

**Table 1:** The five technical areas and eighteen indicators for One Health under the IHR Monitoring and Evaluation Framework

Name of Technical Area	Number of Indicators	Number of Activities
Workforce development	3	16
Real time Surveillance systems	4	29
National Laboratory systems	4	26
Emergency response	4	12
Zoonotic diseases	3	13

### Data collection procedures

Data were collected through desk review of reports (document analysis) namely 2016 Joint External Evaluation report, National Action Plan for Health Security Airtable, 2020 NAPHS review report, state party annual reports (SPAR), ministerial policy statements and sector reports. Additionally, five focus group discussions were done with technical officers from MDAs in relation to the five technical areas until saturation was attained. This was moderated by the principal author (Dr. Herbert Bakiika) who is the Senior Technical Advisor One Health. During desk review, we collected activity reports and strategic documents from the four different line ministries.

### Data analysis

Quantitative data about activity implementation was summarized as frequencies and proportions. Qualitative data following focus group discussions of key informants were transcribed, summarized, key outputs per technical area compiled and thematic analysis done in a formal structured way. The data were analyzed for content to identify emerging themes, and under *a priori* themes (the five technical areas selected to assess the contribution of One Health), then triangulated to synthesize the findings. Noteworthy, this study analyzed the prioritised activities for year one in each of the five technical areas.

## Results

The findings of this study are written under eight sections, including the five technical areas: Zoonotic diseases, national laboratory systems, work force development, real-time surveillance and emergency response operations. The study also reports about three additional areas of governance structures, multisectoral communication and capacity building for the One Health approach. There were 43 key resource persons who participated in this consensus process, from Government of Uganda MDAs in Uganda (n = 25), implementing (n = 10) and development partners (n = 8). (Table 2).

**Fig. 1:**
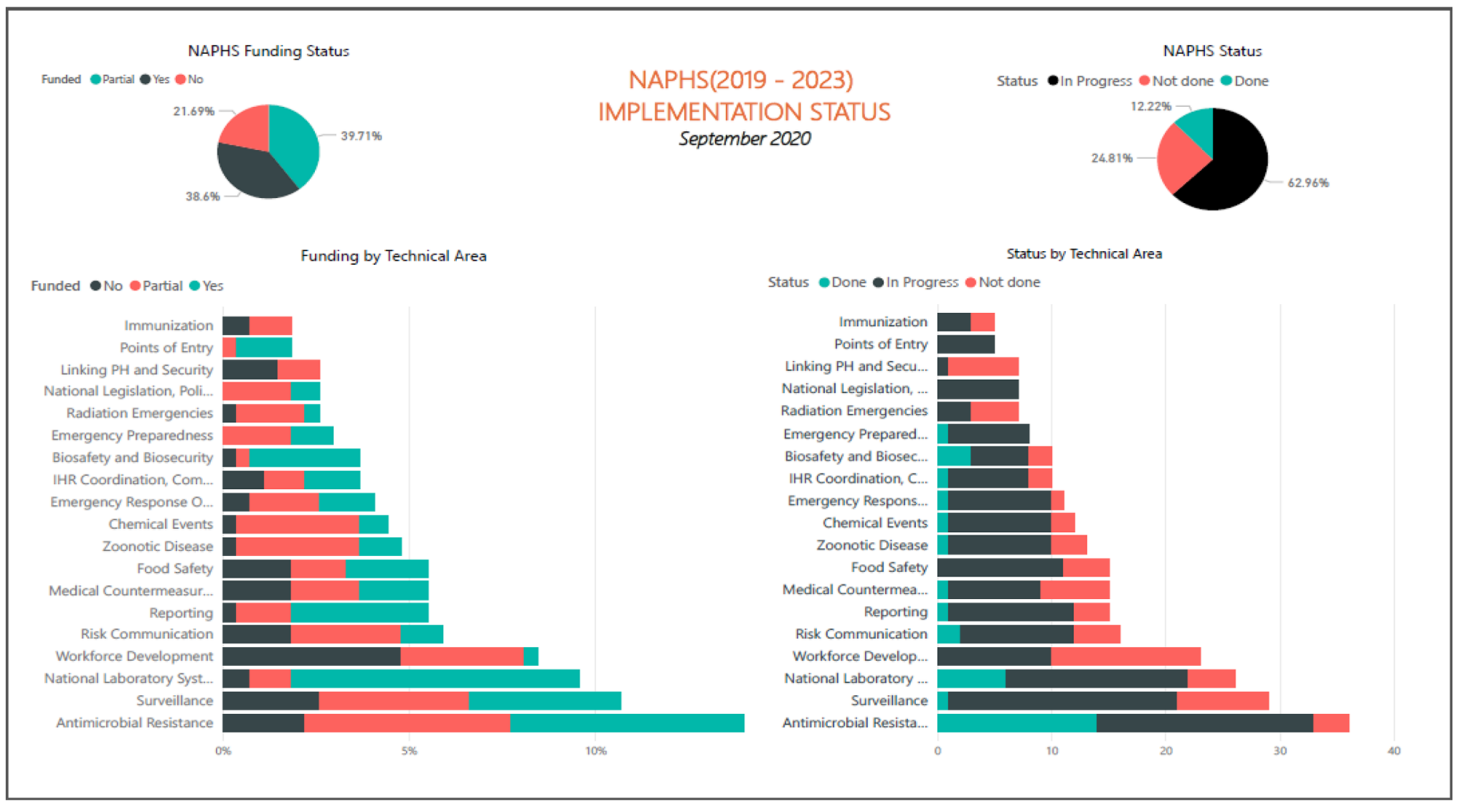
Implementation and funding status of NAPHS 2019–2023 five technical areas zoonotic diseases, national laboratory systems, surveillance, workforce development and emergency response

Although, there were priority areas for year one, majority of NAPHS activities were implemented to varying extents, most of which were still in progress (63%) or accomplished (12.2%). A substantial proportion of NAPHS activities (24%), were not started at all. Most activities were funded (78.3%), with equal distribution between partial (38.6%) and full funding (39.7%). However, specific details of implementation and funding progress are in Fig. 1, below.

**Fig. 2:**
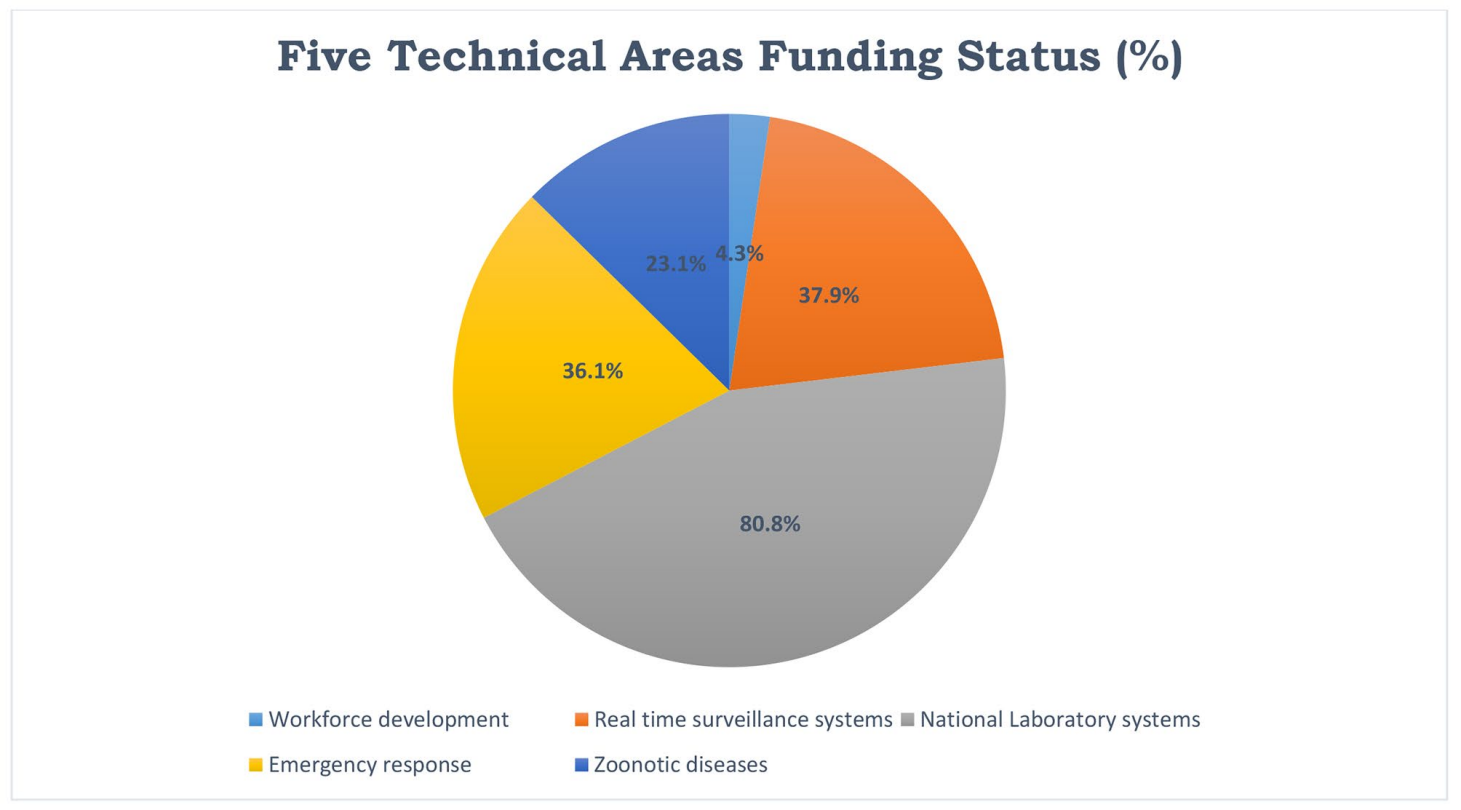
Showing funding status (%) in five technical areas namely; Workforce development, Emergency response, Zoonotic diseases, Real time surveillance systems and National laboratory systems across all sectors

In addition, the five technical areas assessed in this study namely Zoonotic diseases, National laboratory systems, Work force development, Real-time surveillance and Emergency response operations indicated an average funding status of 36.5%, most of which were still in progress (65%) or accomplished (8.6%). A minimum proportion of NAPHS activities (14.2%), were not started at all. Moderate number of activities were funded (36.5%), with closely equal distribution with partially funded activities (40.6%) and not funded activities (22.8%) as indicated in in Table 3. However, specific details of funding progress are in Fig. 2, below.

### Governance structures for the One Health approach

Overall, 100% or thirty-six One Health quarterly and other meetings of One Health Technical Working Groups (OHTWG) have been conducted. This specific assessment considered meetings from establishment of the National One Health Platform in 2016, three years before the NAPHS was launched. These were all the 16 quarterly meetings. Another 20 meetings included 2 high level Annual Directors Meetings, 4 Annual World One Health Day, 4 Annual World Antimicrobial Awareness Week, 4 Annual World Rabies Day and 2 Annual Antimicrobial Conference and 4 Priority Zoonotic Disease Outbreak Incident Management Team meetings.

The rest of this section describes the evolution of One Health institutional frameworks for coordination and collaboration in Uganda. Briefly, in 1980 Uganda established a veterinary public health division within the ministry of health (MoH) hence recognizing importance of integrating animal health [16]. In February 2013, the Uganda Medical Association and Uganda Veterinary Association organized the first in-country One Health conference officiated by the Minister of Health.

**Fig. 3:**
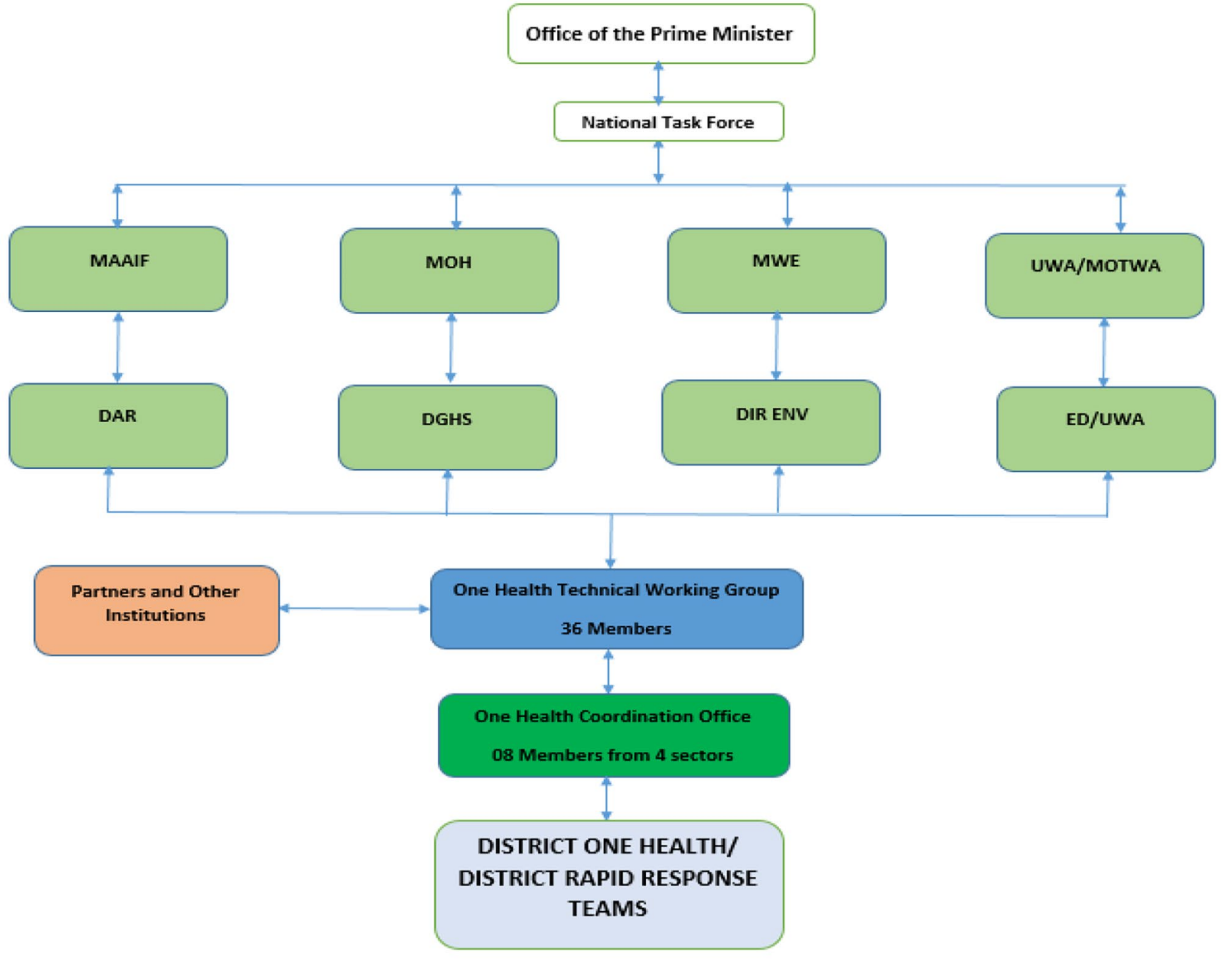
Showing multisectoral coordination, collaboration, and communication across all sectors

This was followed by a One Health framework that was developed in March 2016 by the four line and respective directors; Ministry of Health (MOH) under Director General of Health Services (DGHS), Ministry of Agriculture, Animal Industry and Fisheries (MAAIF) under the Director Animal Resources (DAR), Ministry of Water and Environment (MWE) under the Director Environment Affairs (DEA) and Uganda Wildlife Authority (UWA) under the Executive Director (ED) effectively establishing the national one health platform in 2016 (Fig. 3) [17]. In 2017, the country developed and launched a five year national One Health strategic plan in 2018–2022 that prioritized seven zoonotic diseases; rabies, viral hemorrhagic fevers, anthrax, brucellosis, plague, zoonotic influenza viruses and African Trypanosomiasis [18].

The assessment found an established coordination mechanism from the Office of the Prime Minister, together with development partners, to sub-county level. Noteworthy, the integrated disease surveillance response (IDSR) guidelines indicated that resident district commissioners lead multisectoral outbreak preparedness and responses at district level, through the district epidemic preparedness and response committee.

The 2019 Ebola outbreak in DRC, provided an opportunity for the Infectious Diseases Institute’s ‘One Health’ team to activate district task force committees in West Nile districts that conducted regular planning meetings, participation in the interagency and inter-sectoral meetings with partners in refugee health. The district epidemic committees were converted to One Health teams led by the Resident District Commissioners. Sub-county HIV/AIDS committees supported creation of sub county One Health teams funded by Presidential Emergency Plan for AIDS Relief (PEPFAR).

**Table 2:** List of participating organizations in the assessment of the contribution of the “One Health” approach to strengthening health security in Uganda

Stakeholder	Organization Name	Attendees
Government departments in Uganda	Members or participants of the national one health platform (NOHP). These were government (a) ***Ministries***: Office of the Prime Minister, Ministry of Health (MoH), Ministry of Agriculture, Animal Industry and Fisheries (MAAIF), Ministry of Water and Environment (MWE), Ministry of Tourism, Wildlife and Antiquities (MoTWA), Ministry of Science and Technology (MoSTI), Ministry of Defence and Veteran Affairs (MoDVA), Ministry of Security (MoS) (b) ***Departments/Agencies***: Uganda Wildlife Authority (UWA), National Institute of Public Health, Central Public Health Laboratories, Uganda National Council of Science and Technology, Uganda Police Force, National Animal Disease Diagnostics Epidemiological Center	25
Implementing partners	Infectious Disease Institute, Tackling Deadly Diseases Africa program, Uganda Red Cross, Uganda Medical Association, Baylor Uganda, Africa One Health University Network, Africa Field Epidemiology Net Work, Makerere University School of Public Health	10
Development partner	World Health Organization, World Organisation for Animal Health, United Nations Food and Agricultural Organisation, Center for Disease Control and Prevention, United States Agency for International Development, UN environment, International Office of Migration.	08

### Improved multisectoral communication

Uganda launched its national One Health risk communication strategy in January 2020 and disseminated it to key stakeholders [19]. The four-line ministries officially nominated national IHR focal persons that support disease reporting and progress in implementation of NAPHS to achieve health security. During Marburg outbreak 2017, members of parliament were engaged in risk communication and social mobilization to create awareness among the general public in their constituencies.

Previously, three international conferences were held between 2013 and 2019 by the Africa One Health University Network (AFROHUN) formerly One Health Central and Eastern Africa (OCHEA). These conferences were in Addis Ababa, Ethiopia in 2013 and in Kampala in November 2015 and July 2019 under the themes, *“One Health and the Control of Infectious Diseases: Building Capacity, Systems and Engaging Communities”*, *“Strategic approach to Global Health Security through One Health Innovation: Vision 2035”* and *“Harnessing One Health for Global Health Security”*, respectively. Headquartered in Kampala, OCHEA was present in six countries of Ethiopia, Democratic Republic of Congo, Kenya, Tanzania, Rwanda and Uganda, with partners in USA: The University of Minnesota and Tufts University.

### Capacity building for one health

`This involved several initiatives including in-service training and fellowships. The Africa One Health University Network (AFROHUN) was established from the One Health Central and East Africa initiative (OHCEA). AFROHUN is an international network of institutions of higher learning located in sixteen universities in eight countries in Eastern, Central and Western African region, including Uganda. AFROHUN partners with the University of Minnesota and Tufts University in the USA. Eighty AFROHUN students were enrolled into the One Health Institute in April 2018. Additionally, AFROHUN enrolled technical officers in One Health field fellowship in Eastern Uganda including the Rift Valley Hemorrhagic Fever talk.

The line ministries of health, water and environment, agriculture, animal industry and fisheries, and tourism developed training materials for district One Health teams at sub-national level including a curriculum. Resolve to Save Lives and the Global Health Security project at the Infectious Diseases Institute trained district One Health Teams in 24 out of 134 districts of Uganda (17.9%). This training focused on strengthening sub-national multisectoral coordination, collaboration and communication during emergency preparedness and response.

Finally, in-service training was done for regional veterinary laboratories and National Animal Disease Diagnostics and Epidemiology Centre (NADDEC) in sample collection guidelines.

### Prevention of priority zoonotic diseases

Prevention of priority zoonotic diseases was in progress for four of the five priority activities for year one (69%). Two (23.1%) of the four were fully funded while the two (69.2%) were partially funded as indicated in Table 3.

The national surveillance system in the ministry of agriculture was strengthened by recruitment of surveillance officers under the supervision of the District Veterinary Officers, establishing an e-mail contact on which to send alerts was shared with stakeholders and an animal health information system that uses cell phones for data entry was developed.

The National One Health coordination office was facilitated with salary, transportation, communication enhancement for the national One Health platform coordinator. A television set to relay Key Performance Indicators, workstations and facilitation for quarterly coordination meeting was provided. One Health training materials were developed and disseminated to 21 districts namely Nakaseke, Kiryandongo, Kisoro, Kanungu, Lyantonde, Luwero, Kasese, Kumi, Kyotera, Agago, Amuru, Kiboga, Mbale, Lamwo, Kotido, Kitgum, Nebbi, Kween, Tororo, Busia and Nakasongola.

**Table 3:** Performance of One Health in the five technical areas

Name of Technical Area	Funding Status (%)			Implementation Status (%)		
	Funding	Partial	None	Done	Not Done	In progress
Workforce development	4.3	39.1	56.5	0	35	43
Real time surveillance systems	37.9	37.9	24.1	3	24	69
National Laboratory systems	80.8	11.5	7.7	23	4	62
Emergency response	36.4	45.5	18.2	9	0	82
Zoonotic diseases	23.1	69.2	7.9	8	8	69

### Strengthened national laboratory systems to enhance detection capabilities

About 81% of activities were fully funded and 11.5% partially funded. Of the planned activities, 23% were completed, while implementation was in progress in 61.5% of the activities as indicated in Table 3. Using the One Health approach, ten regional veterinary laboratories and the animal health national reference laboratory (NADDEC) were assessed by FAO for capacity to conduct zoonotic disease diagnostics. All the ten regional veterinary laboratories were capable of running enzyme linked immunosorbent assays (ELISA) tests. Further, Arua and Mbarara regional veterinary laboratories were integrated into the national specimen referral and transportation network. In fact, between January to March 2020, fifteen antimicrobial resistance isolates were transported to the Uganda National Health Laboratories Services (UNHLS) from Mbarara veterinary laboratory.

This captured quality assurance activities including participation in External Quality Assessment exercises. The Uganda National Health Laboratory Services participated in an external quality assurance scheme (PT-EQA-CAP). Indeed, external quality assurance panels were sent to Mbarara veterinary laboratory from UNHLS on a quarterly basis. Arua Regional Referral Hospital progressed from ‘one star’ to ‘four stars’ and the animal health laboratories (NADDEC) and college of veterinary medicine and biosecurity (COVAB) both progressed from ‘0 star’ to ‘one’ and ‘two stars’ respectively. There were improvements in quality management through revising the guidelines for implementation of the national external quality assessment program; provision of supplies to UNHLS; implementing the national Gram, identification and antimicrobial susceptibility testing (AST) proficiency testing scheme at eighteen human and two animal health surveillance sites.

Governance aspects of the laboratory systems were strengthened through development and implementation of thirteen laboratory standard operating procedures (SOPs) on pre-analytical, analytical and post-analytical stages for microbiology at seven human and three animal surveillance sites. Not least, a strategic plan for animal health laboratories was developed.

### Work force development pillar

Overall, 23 activities were planned on workforce development and only one (4.3%) had full funding during the past one year of NAPHS implementation. Implementation was in progress in ten (43%) of the 23 activities as indicated in Table 3.

There were several human capital capacity building initiatives that emphasized One Health. Generally, the IHR focal persons were trained on their roles by WHO. Specifically, the Africa Field Epidemiology Network Advanced Field Epidemiology Training Program (AFENET) reserved a quota for about two to three veterinarians annually during their training. In addition, the Master of Public Health program at the School of Public Health, Makerere University Kampala enrolled over 30% veterinarians to reinforce One Health workforce development. Also, FAO incorporated One Health into in service veterinary training (ISAVET). Not least, the AFROHUN One Health fellowship and residency programs were ongoing during this period.

Finally, with support from AFENET, a database of all the trainees in public health fellowship programs and the school of public health master’s graduates was put in place as part of strategic information establishment.

### Real time surveillance pillar

Overall, surveillance had 29 summary activities from which full (37.9%) and partial funding (37.9%) was available for 11 respectively. Twenty activities were being implemented and one (3%) had been completed as indicated in Table 3. There were efforts to enhance animal health surveillance as animal bites and zoonotic illness such as influenza like viruses were incorporated in the routine ministry of health weekly epidemiological bulletin. Further, animal events reporting is now captured in the electronic integrated disease surveillance and response (e-IDSR), hence strengthening event-based surveillance. Noteworthy, a reporting system called animal resources information systems (ARIS) from Africa Union Inter African Bureau for Animal Resources (AU-IBAR) was established within the ministry of agriculture to quicken the reporting of events.

Training efforts to orientate the workforce about these One Health changes were done using version three of the IDSR by WHO-Afro, in preparation for rollout. This revised version had a module for animal diseases surveillance and was launched in Uganda in August 2021. Indeed, event-based surveillance training for national teams was conducted using the epidemic intelligence in open sources system (EIOS). Twenty personnel were trained on in-service applied veterinary epidemiology and the veterinary team of West Nile were trained on animal diseases notification system of the short message service (SMS) coded 6767.

### Emergency response operations pillar

Overall, 36.4% of the summary activities under emergency response operations technical area was funded and out of the eleven activities, partially (45.5%) whilst two (18.2%) not funded as indicated in Table 3. About one in five (17.9%) of the districts established district One Health teams at sub-national level to strengthen multisectoral emergency preparedness and response with the goal of containing diseases at source.

Joint emergency response plans against key prioritized zoonotic diseases (PZDs) namely Anthrax and Rift Valley Fever were developed. In fact, joint Outbreak response were conducted by all sectors in recent outbreaks of Rift Valley in Kabale in 2020 and Crimean Congo Hemorrhagic Fever in Lyantonde.

With support from the Infectious Disease Institute (IDI), national and subnational Emergency Operations Center (EOC) public awareness trainings were conducted in National One Health platform and Southwestern regions focusing on Public Health Emergency Operations Center (PHEOC) operations.

## Discussion

### Key findings

This study identifies key learnings. First, the study demonstrated that a One Health framework was used to implement multiple activities in the National Action Plan for Health Security. Secondly, the National One Health Platform approach strengthened Uganda’s outbreak detection, preparedness, and response for prioritized zoonotic diseases through effective multisectoral coordination and synergies decentralized to sub-national levels. Third, this study identified the funding areas for investment. Despite limited funding it was feasible to implement a substantial proportion of One Health activities in Uganda. Fourth, collaboration with international development partners was vital in integrating and sustaining the One Health concept in human capital development programmes at universities.

### Findings in relation to existing literature

Previous research work by Zumla and colleagues (Zumla, 2016) documented the One Health approach as an important component of the Global Health Security Agenda, and in fact termed this as “One Health Security”. That, despite the numerous bottlenecks working across conflicting cultures, multiple sectors and multidisciplinary professionals, the One Health approach is necessary to prevent, detect and appropriately respond to public health threats of concern [20]. Indeed, turning the tide against the MERS-CoV and Ebola outbreaks in the middle east and west Africa respectively presented unique opportunities for the One Health approach [20].

Country level assessments are crucial in identifying areas of improvements. Rosque and colleagues’ analysis of national bridging workshops (NBW) activities and collaborative reported that countries overestimated their capacities at the human–animal–environment interface [21]. Munyua and colleagues conducted a 10-year assessment of the One Health approach in Kenya that yielded similar findings as our study [22]. There were substantial improvements with establishment of the Zoonotic Diseases Unit (ZDU), that coordinated multisectoral efforts, with support from international development partners. Field Epidemiologists were trained with incorporation of Veterinary Professionals, *inter alia*, that contributed to effective zoonotic disease prevention detection and response such as the Rift Valley Fever. The challenges of sustainability and funding priorities were consistent to those of our study. These challenges were augmented by a multi-African country assessment by Fasina and colleagues [23] and a systematic review by Ribeiro and colleagues [24]. Fasina and Ribeiro argue that sustainability of One Health will be hinged on inclusion of a theory of change, monitoring and evaluation frameworks, and tools for standardized evaluation of One Health policies. Further, the authors proposed aiming at outputs and outcomes-driven approach would be more yielding than an activity-driven approach.

In its white paper, the 5th International One Health Congress in Saskatoon, Canada, in June 2018 suggested evolution of science into a One Health approach to improve health and security [25]. The backdrop of this argument was an exponential increase in priority zoonotic diseases between 2000 and 2018, such as H5N1, H1N1, H7N9, MERS-CoV, SARS-CoV, Ebola, Zika, Rift Valley Fever amongst others. This paper estimated that 60% of emerging infectious diseases had animal sources; and emerged at a rate of one in eight months. Indeed, the WHO has gone ahead to form multidisciplinary platforms such as Technical Advisory Group on SARS-CoV-2 Virus Evolution (TAG-VE). In Uganda, the National One Health Platform has continued to perform this function with proposals to update curriculums of higher institutions of learning to capture the one health lens. In fact, a recent study in Uganda demonstrated how One Health was integrated into practical learnings in health training at Makerere University [26]. However, workforce trainings ought to overcome the infectious diseases bias.

Collaborative development and implementing partners played a key role in Uganda’s successful One Health approach. Smith and colleagues [27]) previously (in 2015) reported limited implementation of One Health activities due to challenges of Uganda’s decentralization policy. Additional challenges were international, external actors not engaging with the Ugandan state; actors setting up parallel structures and activities; actors deciding when emergencies begin and end without consultation; weak Ugandan state capacity to coordinate its own integrated response to disease and limited collaboration between core Ugandan planning activities and a weak, increasingly devolved district health system. This study reports several of these challenges were circumvented, by emphasizing a collaborative platform for joint action by all stakeholders, forming district One Health teams, training them and supporting their activities.

### Strengths and limitations of the study

This study had several strengths and few shortcomings. This is one of the first papers to document the contribution of the One Health approach to national health security in sub-Sharan Africa. Furthermore, the participants were representative with a broad reach of subject matter experts in a multisectoral approach of engaging stakeholders from ministries, departments, agencies and partners. Also, this assessment was comprehensive covering all the five technical areas, eighteen indicators and ninety-six activities. Although the data collection was retrospective and likely to be limited by recall bias, this was overcome through document analysis, probing, member checking, validation, and triangulation of findings to improve the trustworthiness. Finally, the paper does not report on the two key One Health technical areas of biosafety and biosecurity, and antimicrobial resistance.

### Implications for health security policy and practice

There were several policy relevant implications. Low- and middle-income countries could consider multisectoral collaboration and coordination via National One Health Platforms to implement Health Security and the IHR 2005. Uganda’s NOHP platform provided an effective channel for communication and resolving implementation considerations enabling sub-national action. This study also compared progress of implementation to funding gaps, and this highlighted investment options for country governments and development partners. Third, using the one health lens to make this assessment demonstrated a self-driven accountability by the key actors to the public and funders. Not least, using the online Airtable© real time monitoring system of NAPHS implementation, the funded and unfunded activities were identified for reprioritization. This prioritization process involved multisectoral stakeholders to develop the following years operational plan.

### Potential areas for future research

This study was a process evaluation and thus leaves room for further investigation. Conducting an impact evaluation prospectively would delineate the link between the One Health approach as intervention, and important outcomes such as timely detection and control of specific disease outbreaks would be informative. Additionally, documenting costs and cost-effectiveness of the One Health approach would inform decision making in low-income countries of sub-Saharan Africa. Subsequent papers could assess and report on the two key one health technical areas of biosafety and biosecurity, and antimicrobial resistance missed here.

### Conclusions from the study findings

This study demonstrated the integral aspects of One Health implementation in strengthening Uganda’s health security. Establishing a National One Health Platform strengthened multi-sectoral collaboration, communication and coordination which accelerated implementation of the National Action Plan for Health Security to sub-national levels. Implementation of multiple National Action Plan for Health Security activities heavily relied on investment in funding gaps, by the state and non-state actors, which reinforced Uganda’s health security to achieve the International Health Regulations 2005 and the Global Health Security Agenda.

## Data Availability

All data generated or analyzed during this study are included in this published article. The data sources were majorly documenting of the Government of Uganda including strategic plans, annual performance reports, field reports and archives found in the various libraries of ministries, departments and agencies.
